# The Significance of IDH1 Mutations in Tumor-Associated Seizure in 60 Chinese Patients with Low-Grade Gliomas

**DOI:** 10.1155/2013/403942

**Published:** 2013-11-13

**Authors:** Ruofei Liang, Yingjun Fan, Xiang Wang, Qing Mao, Yanhui Liu

**Affiliations:** Department of Neurosurgery, West China Hospital of Sichuan University, Chengdu 610041, China

## Abstract

*Background*. Seizure is a common clinical presentation in patients suffering from primary brain tumors, especially from low-grade gliomas (LGGs). However, the genetic factors of tumor-associated seizure, at present, are still very poorly understood. The aim of this study was to investigate the potential correlation between tumor-associated epilepsy and IDH1 mutations in a Chinese population with LGGs. *Materials and Methods*. This study reviewed 60 patients with histologically confirmed low-grade gliomas, and the status of IDH1 was detected after the operation at our institution. Univariate and multivariate logistic regression analysis were used to explore the potential risk factors for tumor-related seizures. *Results*. IDH1 mutation was detected in 46 (76.7%) patients, among which 14 patients had no epilepsies and 32 patients had epilepsies (*P* = 0.023, chi-square test). Multivariate logistic regression analysis demonstrated that the mutation of IDH1 seems to be the strongest predictor for preoperative seizure (OR, 6.130; 95% CI, 1.523–24.669; *P* = 0.011). *Conclusions*. IDH1 mutation was frequently detected in LGGs, and it may result in tumor-related seizures.

## 1. Introduction

Seizure is a common clinical presentation in patients suffering from primary brain tumors, especially from low-grade gliomas (LGGs). Around 60% to 95% of all patients with LGGs present with seizures during their life [1–10], but the incidence of seizures is variable, despite the fact that the histology features and the location of tumors are similar. In recent research, authors have shown that patients with the histological hypotype of oligodendroglioma and oligoastrocytoma are more prone to having epilepsy than those with astrocytomas [8]. Patients with tumors that are involved in the regions of temporal lobe are more likely to suffer from seizure [8, 10–12]. However, at present, the genetic factors of tumor-associated seizures are still very poorly understood. Mutations in the enzyme cytosolic isocitrate dehydrogenase 1 (IDH1) are found in more than 68% of WHO grade II and III gliomas and secondary glioblastomas (GBM) in human brain cancers [13–15]. The aim of this study was to investigate the potential correlation between tumor-associated epilepsy and IDH1 mutation in a Chinese population with LGGs. In this retrospective study, a series of 60 consecutive LGGs were included, which consist of 19 oligodendrogliomas (WHO grade II), 19 oligoastrocytomas (WHO grade II), and 22 astrocytomas (WHO grade II) diagnosed by histopathology.

## 2. Subjects and Methods

### 2.1. Materials and Methods

#### 2.1.1. Patients and Tissue Specimens

Patients who underwent a primary resection between August 2011 and December 2012 were included in this study, and the status of IDH1 was detected after the operation in our institution. Patients who underwent secondary resection and those with spinal and infratentorial lesions were excluded from our study. The data collected included the age, gender, histology of the tumor, location of the tumor, and the profile of seizures. The assessment of seizure type was performed by two neurosurgeons independently. 

#### 2.1.2. Pathologic Review

All gliomas samples were processed in the department of pathology at our institution. They were assessed and graded independently by two neuropathologists based on the 2007 WHO guidelines [16]. Cases in controversy were reexamined by the whole pathologist team in the department of pathology until a consensus was reached.

#### 2.1.3. The Status of IDH1

Direct sequencing was performed by Invitrogen Life Technologies (Shanghai, China).

#### 2.1.4. Tumor Location

The anatomical location was determined based on the evaluation of radiological records and operation notes. The locations of the gliomas were as follows: frontotemporoinsular lobe (*n* = 3), occipitoparietal lobe (*n* = 3), frontoparietal lobe (*n* = 4), temporoinsular lobe (*n* = 1), parietotemporal lobe (*n* = 2), frontotemporal lobe (*n* = 7), occipital lobe (*n* = 1), parietal lobe (*n* = 7), temporal lobe (*n* = 14), and frontal lobe (*n* = 18). In order to facilitate the statistical analysis, tumors located in the temporal lobe were assigned in the group of temporal tumors, and the others were assigned in the group of nontemporal tumors.

#### 2.1.5. Statistical Analysis

SPSS software (version 21.0) was used for statistical analysis. Statistical analysis for dichotomous variables was performed using the chi-square test, and continuous nonparametric data was performed using the Mann-Whitney *U* test. The mutation of IDH1, tumor location, and histology were selected for multivariate logistic regression analysis based on our clinical experience and prior hypothesis. A probability value (*P* value) of 0.05 or less was treated as statistical significance.

## 3. Results 

A total of 60 patients were included in this study during the above-mentioned period. The clinical information of the 60 patients was listed in the Table 1. There were thirty-four males and twenty-six females (male-female ratio, 1.3) ranging from 17 to 65 years old (mean, 39.5 years). Thirty-seven (61.7%) patients presented with epilepsy at the onset of disease, whereas 23 (39.2%) patients had no preoperative seizures. Among the noneseizure group, 13 patients presented with headache or dizziness, 6 patients presented with focal neurological deficits, 2 patients were incidental cases, and 1 patient was admitted via the emergency room. Patients with preoperative seizure were younger than those without preoperative seizure (mean 38 versus 44 years; *P* = 0.05, Mann-Whitney *U* test). Of the 37 patients with seizures, 24 (64.9%) patients had experienced secondary generalized seizures while 13 (35.1%) had partial seizures. However, there were no significant differences in tumor histology, tumor location, and gender.

Patients with IDH1 mutation and without this alteration had about the same mean age (38.5 versus 47.5 years; *P* = 0.256, Mann-Whitney *U* test). IDH1 mutation was found in 46 (76.7%) patients. Of the 23 patients without epilepsies, 14 cases (60.9%) had IDH1 mutation, whereas 32 cases were found to have IDH1 mutation among the 37 epileptic patients (86.5%) (*P* = 0.023, chi-square test). The mutation was also discovered in 10 of the 13 (77.0%) patients who had partial epilepsies, while the same mutation was found in 20 of the 24 (83.3%) patients who suffered from secondary generalized epilepsies. No significant correlation was observed between the types of epilepsy and IDH1 mutation (*P* = 0.678, Fisher, *s* Exact test). Multivariate logistic regression analysis demonstrated that the mutation of IDH1 seems to be the strongest predictor for preoperative seizure (OR, 6.130; 95% CI, 1.523–24.669; *P* = 0.011; Table 2). However, significant differences in the factors of tumor histology and tumor location were not found. 

## 4. Discussion

The etiology of tumor-related seizures is multifactorial and still very poorly understood [17, 18]. The cells of the astrocytic tumor have the ability to generate action potentials [19, 20], which could be the origin and spreading route of seizure activity. Some scholars reported that the cells of glioma could release glutamate [21, 22], which could kill the peritumoral neurons through an excitoxic mechanism. Glutamate has also been detected in brain tumor specimens from patients with active epilepsy [23]. These findings suggested the importance of glutamate in the generation of tumor-related seizures. The mutations of nicotinamide adenine dinucleotide phosphate- (NADP-) (+) dependent IDH1 were frequently identified in WHO grade II or III gliomas [13]. Mutant IDH1 could directly produce 2-hydroxyglutarate (2HG) from *α*-ketoglutarate (*α*KG), which caused 100-fold increase of 2HG level in tumors with IDH1 mutation [24]. 2HG is structurally similar to glutamate, which is able to activate N-methyl-d-aspartate (NMDA) receptor [25]. This may suggest that 2HG was involved in the epileptogenesis. 2HG dehydrogenase deficiency could result in an accumulation of the metabolite D-2HG which causes seizures [26].

To identify the possible association between seizure occurrence and IDH1 mutation, we have retrospectively reviewed a total of 60 Chinese patients with LGGs, including 19 oligodendrogliomas, 19 oligoastrocytomas, and 22 astrocytomas. We demonstrated a correlation between IDH1 mutation and epileptic seizures (*P* = 0.023, chi-square test), which echoed with the results (*P* = 0.001, chi-square test) reported by Stockhammer et al. [27]. In our findings, the frequency of IDH1 mutations in LGGs is 76.7%, which correlated with previous studies [13–15]. The lack of statistical significance in the age of the groups with and without IDH1 mutation was also consistent with previous findings [15]. In our study, 61.7% of the patients with LGGs were admitted with epilepsy as the initial symptom, which was similar to that previously reported of 60%–95% rate [1–10]. In fact, seizure was the most common reason for neuroimaging leading to the diagnosis of gliomas. Generally, the high occurrence of epilepsies in LGGs may indicate the slowed growth of tumor, and a longer disease course may contribute to the generation of epilepsy [6]. 

In our findings, the younger patients with LGGs were more likely to suffer from seizures, which complied with previous studies [4, 12]. This was probably because younger patients with less developed brains were more susceptible to epileptogenic activity than older patients [12]. In contrast to other early studies [8, 10–12], we did not find that patients with gliomas of the temporal lobe and the oligodendroglial type (including oligodendroglioma and oligoastrocytoma hypotype) were more prone to suffering from epilepsies. The negative results may be, to some extent, due to the different standard of pathologic diagnosis in different institutions. 

However, the limitations of this study should be acknowledged. Firstly, this is only a retrospective and correlative study. Secondly, it is possible that our samples may not represent the entire glioma population due to the small number of cases in this study. Thirdly, the possible association between the tumor-associated epilepsy and the survival time of patients with LGGs was not performed because the duration of follow-up in our study was so short that it could not be statistically analyzed. This remains to be carried out in future research. Lastly, the presence of IDH1 mutations was often noted in LGGs, but this may be just an epiphenomenon, with regard to the presence of seizures. It is quite possible that they may not be directly related. So, experiments on animals should be carried out in further research. In conclusion, the current study provided evidence that IDH1 mutation was frequently detected in LGGs, and IDH1 mutation may result in tumor-related seizures.

## Figures and Tables

**Table 1:** Clinical information of patients (*n* = 60).

Characteristic	Number	Value (%)
Gender		
Male	34	56.7
Female	26	43.3
Tumor location		
Nontemporal	33	55.0
Temporal	27	45.0
Presenting symptom		
No seizures	23	38.3
Seizures	37	61.7
Partial	13	21.7
Generalized	24	40.0
IDH1 status		
Mutated	46	76.7
Wild-type	14	23.3
Histology		
Astrocytoma	22	36.7
Oligoastrocytoma	19	31.7
Oligodendroglioma	19	31.7

**Table 2:** Multivariate logistic regression analysis.

Variable	OR	95% CI	*P* value
Mutation of IDH1	6.130	1.523–24.669	0.011
Tumor location	1.829	0.573–5.836	0.308
Histology	0.583	0.177–1.919	0.374

OR: odds ratio; CI: confidence interval.
